# Oxygen Transport and Stem Cell Aggregation in Stirred-Suspension Bioreactor Cultures

**DOI:** 10.1371/journal.pone.0102486

**Published:** 2014-07-17

**Authors:** Jincheng Wu, Mahboubeh Rahmati Rostami, Diana P. Cadavid Olaya, Emmanuel S. Tzanakakis

**Affiliations:** 1 Department of Chemical and Biological Engineering, Tufts University, Medford, Massachusetts, United States of America; 2 Department of Chemical and Biological Engineering, State University of New York at Buffalo, Buffalo, New York, United States of America; University of Catania, Italy

## Abstract

Stirred-suspension bioreactors are a promising modality for large-scale culture of 3D aggregates of pluripotent stem cells and their progeny. Yet, cells within these clusters experience limitations in the transfer of factors and particularly O_2_ which is characterized by low solubility in aqueous media. Cultured stem cells under different O_2_ levels may exhibit significantly different proliferation, viability and differentiation potential. Here, a transient diffusion-reaction model was built encompassing the size distribution and ultrastructural characteristics of embryonic stem cell (ESC) aggregates. The model was coupled to experimental data from bioreactor and static cultures for extracting the effective diffusivity and kinetics of consumption of O_2_ within mouse (mESC) and human ESC (hESC) clusters. Under agitation, mESC aggregates exhibited a higher maximum consumption rate than hESC aggregates. Moreover, the reaction-diffusion model was integrated with a population balance equation (PBE) for the temporal distribution of ESC clusters changing due to aggregation and cell proliferation. Hypoxia was found to be negligible for ESCs with a smaller radius than 100 µm but became appreciable for aggregates larger than 300 µm. The integrated model not only captured the O_2_ profile both in the bioreactor bulk and inside ESC aggregates but also led to the calculation of the duration that fractions of cells experience a certain range of O_2_ concentrations. The approach described in this study can be employed for gaining a deeper understanding of the effects of O_2_ on the physiology of stem cells organized in 3D structures. Such frameworks can be extended to encompass the spatial and temporal availability of nutrients and differentiation factors and facilitate the design and control of relevant bioprocesses for the production of stem cell therapeutics.

## Introduction

Realization of the therapeutic potential of pluripotent stem cells (PSCs) including embryonic stem cells (ESCs) and induced pluripotent stem cells (iPSCs) hinges on the development of platforms for large-scale PSC expansion and directed differentiation. The culture of PSCs has been demonstrated in laboratory scale stirred-suspension bioreactors (SSBs) as cell aggregates [1] or on various scaffold types [2], [3]. Compared to static culture, SSBs afford higher cell densities and tighter control of process variables leading to more efficient utilization of media and growth factors [1].

Despite the advantages of culturing PSCs as aggregates in stirred-suspension vessels, less attention has been devoted to limitations in the transfer of medium components to and within PSC clusters. Concentration gradients in the bioreactor environment contribute to stem cell population heterogeneity leading to variable responses to self-renewal or differentiation stimuli. Such unevenness is more pronounced for oxygen characterized by low solubility in aqueous solutions and increased rate of consumption by metabolically active stem cells. Oxygen availability affects directly the viability, proliferation and differentiation propensity of stem cells in vivo and in vitro [4]. Cultivated human ESCs (hESCs) are less prone to spontaneous differentiation and chromosomal aberrations at hypoxic (2–5%) than normoxic (21%) O_2_ level without significantly reduced proliferation [5], [6]. Lower O_2_ tension (pO_2_) in culture also predisposes stem cells to commit along particular lineages including endothelial cells [7] and chondrocytes [8] similar to in vivo processes [4]. More details emerge about O_2_ modulation of the activity of stem cell-fate controlling pathways such as the canonical Wnt/β-catenin [9] and Notch cascades [10]. These effects are largely mediated by transcriptional regulators such as the hypoxia-inducible factors (HIF) interacting with a wide gamut of genes including the pluripotency marker POU5F1 (Oct4) [11]. Hence, knowledge of O_2_ profile among stem or progenitor cells is important for cell fate prediction and control.

The distribution of O_2_ was reported for single hESC aggregates under static conditions [12], [13]. The findings from these studies however may not apply directly to aggregate cultures of ESCs in SSBs where the transfer of O_2_ through multiple interfaces complicates the analysis of the O_2_ tension (pO_2_) that each cell experiences. In most small-scale setups, O_2_ is transferred via the gas/liquid interface (headspace aeration) to the culture medium under agitation. From the medium bulk O_2_ fluxes to cells via a boundary layer surrounding each aggregate and a pore network within each cluster. These ultrastructural characteristics of aggregates have also not been considered explicitly to date. And although a steady state assumption simplifies the mathematical framework for O_2_ transfer in stem cell aggregate culture, it may not proper in the case of SSB cultures. This is because the continuous cell proliferation and dynamics of agitation-induced aggregation result in the temporal modulation of O_2_ levels. Furthermore, eliminating time as a variable in the analysis of mass transfer does not allow the calculation of the duration that a particular cell (or fraction) experiences a pO_2_ below a certain threshold. Differences in the “residence time” at a certain O_2_ concentration among cells may be consequential for the differentiation and/or self-renewal of the stem cell population.

Here, the distribution of O_2_ was calculated from experimental data linked with a mathematical model for mouse ESC (mESC) and hESC aggregates cultivated in dishes and spinner flasks. Aggregates cultured in spinner flasks under different agitation rates were analyzed at different time points and ultrastructural attributes such as porosity and tortuosity were determined for the first time. The effective diffusivity of O_2_ within the aggregates and parameters associated with the specific rate of O_2_ consumption were also computed by coupling measurements with a transient diffusion-reaction model. This model was subsequently paired to a population balance equation (PBE) depicting the time evolution of aggregate size distribution of proliferating ESCs under agitation. The multiscale PBE/diffusion-reaction model facilitated the calculation of the O_2_ profile in the medium bulk and inside the aggregates in stirred suspension. Moreover, the fraction of cells experiencing hypoxia was predicted. The computational model described in this study can be utilized in the development of differentiation strategies and the design and control of relevant bioprocesses for the production of stem cell therapeutics.

## Material and Methods

### Experimental methods

#### Embryonic stem cell culture

Human ESCs (H9 cells, WiCell, Madison, WI; passages 30–60) were maintained on dishes coated with Matrigel (BD Biosciences, San Jose, CA) and grown in 5% CO_2_/95% air at 37°C with mTESR1 medium (Stem Cell Technologies, Vancouver, BC) replaced daily. Four hours before harvesting, cells were treated with the Rho-associated kinase inhibitor (ROCKi) Y-27632 (10 µM, EMD Chemicals, Gibbstown, NJ). Colonies were dissociated with Accutase (Innovative Cell Technologies, San Diego, CA) into single hESCs. For static aggregate culture, cells were transferred to poly HEMA-treated (Sigma-Aldrich, St. Louis, MO) Petri dishes with mTESR1. Dispersed hESCs (10^5^/ml) were seeded in 125-ml ProCulture spinner flasks (Corning, Corning, NY) with mTESR1 medium and 10 µM ROCKi. Agitation was kept constant during each experiment and medium was replaced daily thereafter without the addition of ROCKi.

Mouse E14Tg2a ESCs (Mutant Mouse Regional Resource Centers (MMRRC), University of California-Davis, CA) were maintained in defined serum-free medium (DSFM) as described [14] (see *Materials S1*). For stirred-suspension culture [14], mESCs were transferred in DSFM to 125-ml ProCulture spinner flasks at 5×10^4^ cells/ml. The agitation was kept constant (60–100 rpm) throughout each run.

#### Oxygen consumption

Cell aggregate samples of known volumes were transferred to 15 ml-centrifuge tubes and allowed to settle. Without removing aggregates, the medium was replaced by PBS (pH 7.4) containing 0.35 g/L HEPES, 0.5 g/L bovine serum albumin, and 300 mg/dL glucose (Sigma-Aldrich, St. Louis, MO) with a Bunsen solubility coefficient of α_m_ = 1.27×10^−9^ mol/(cm^3 ^mm Hg) at 37°C [15], [16]. The cell suspension was transferred to a 30-ml cylindrical container (2.3 cm wide) with a magnetic cylindrical stirring bar (1.59 cm×0.79 cm). The container was set on a submersible magnetic stirrer plate in a water bath at 37°C bath and agitation was set to 60 rpm. A dissolved O_2_ (DO) polarographic probe (VWR, Bridgeport, NJ) was inserted in the container through the cap with a rubber seal ensuring airtight fitting. DO data (mg/l) were logged on a computer connected to the DO probe/meter. Then, aggregates were transferred to a 96-well plate and images were taken and processed (see below) to obtain the total number of aggregates and pertinent size distributions [17].

#### Stem cell aggregate micrograph acquisition and analysis

Samples of known volume from spinner flasks, dishes or the DO chamber were transferred to a 96-well plate and images from each well were acquired at 4x magnification with a camera (Moticam 2300, Motic, Richmond, BC) connected to the microscope. Raw images were imported into ImageJ (U.S. National Institutes of Health, Bethesda, Maryland, USA, http://imagej.nih.gov/ij/) and for counting total numbers of aggregates. After background subtraction (rolling ball method), the diameter of each aggregate was calculated as the mean of two perpendicular diameters and size distributions were generated [17].

**Figure 1:**
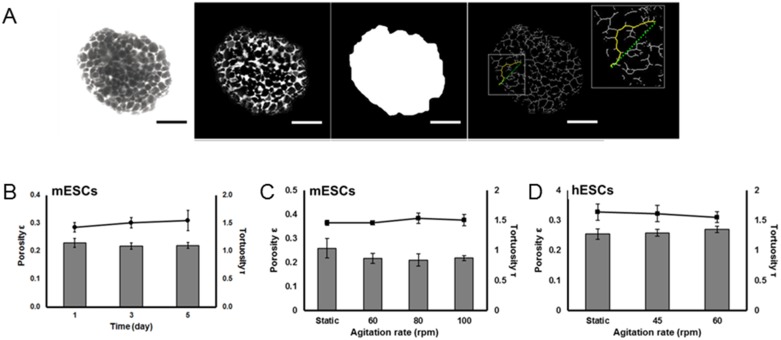
Structural characteristics (tortuosity and porosity) of mESC and hESC aggregates under different conditions. (A) Optical sections were acquired for each aggregate via CLSM and processed to obtained tortuosity and porosity values. For each condition, at least 10 aggregates were imaged by CLSM (30 sections/aggregate) for data analysis. Tortuosity (lines) and porosity (bars) data are shown for (B) mESC aggregates at different time points at 100 rpm, (C) mESC aggregates at different agitation rates on day 3, and (D) hESC aggregates at different agitation rates on day 4. The values in (B–D) are shown as mean±st. dev.

Porosity ε and tortuosity τ were determined in aggregates transferred to culture medium containing 10 mg/ml fluorescein isothiocyanate (FITC)-dextran (MW: 4.4 kDa, Sigma). The aggregate suspension was then transferred to a microscopy slide with a well, sealed with a coverslip and visualized by confocal laser scanning microscopy (CLSM) with a Zeiss LSM 510 Metal NLO system (Zeiss, Thornwood, NY). Aggregates were optically sectioned (10–12 sections per aggregate) at 5-µm steps and the micrographs were processed in MATLAB (Mathworks, Natick, MA) to obtain porosity and tortuosity values (**Fig. 1A**; see *Materials S1*).

#### Statistical Analysis

Data were expressed as mean±st.dev. unless stated otherwise. ANOVA and the *post hoc* Tukey test were performed using Minitab (Minitab Inc, State College, PA) with p<0.05 considered as significant.

### Modeling methods

#### Transient reaction-diffusion model

For the transient reaction-diffusion model employed in this study, the aggregates were assumed to be spherical and thus spherical coordinates were employed to describe the O_2_ concentration (C) profile in each aggregate:
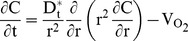
(1)where r is the aggregate radius, t is the time and 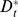 is the effective diffusion coefficient. Taking into account the porosity ε and tortuosity τ, 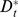 is calculated as
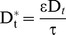
(2)where D_t_ is the diffusion of O_2_ in the tissue (aggregate).

The consumption of O_2_ (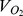
in mol/(cm^3^.s)) was described by Michaelis-Menten (M-M)-type kinetics:
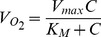
(3)where V_max_ is the maximum O_2_ consumption rate and K_M_ is the M-M constant. Because the system comprises aggregates of multiple sizes, cell clusters were classified into 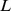 groups or bins. Here, the utilization of 7 bins provided sufficient resolution of the aggregate size distribution. Equation 1 was solved for each group [18] (see *Materials S1*) subject to appropriate initial and boundary conditions.



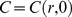
(4)

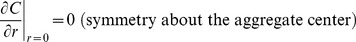
(5)

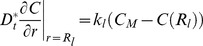
(6)where *R_l_* is the radius for the aggregates in the *l* ^th^ bin. Calculation of the value of the O_2_ transfer coefficient *k_l_* from the bulk liquid to the aggregates was based on the Frössling equation [19] relating the particle Sherwood (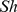) and Schmidt (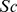) numbers shown in the *Materials S1* section.

The total rate of O_2_ transfer from the bulk to all aggregates (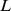 groups) is:
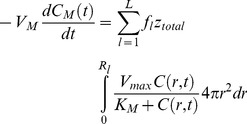
(7)where 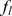 and 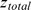 are the fraction of aggregates with average diameter 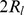 and the total number of aggregates, respectively. Integrals were calculated via the trapezoidal rule using C values from solving Equation 1 and the Euler method was implemented yield estimates of the O_2_ concentration 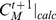 in the medium bulk at the 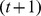-time step in Equation 7.

Values for 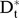, 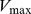 and 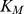 were obtained by minimizing the objective function [20] comparing the calculated values 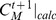 with the experimental ones 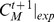 at each time point:
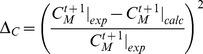
(8)


#### Development of PBE model for the bioreactor culture of ESC aggregates

A size-structured PBE model was employed to simulate the temporal evolution of size of ESC aggregates cultured in spinner flasks [1] considering the contributions of ESC proliferation and collisions between particles to aggregate size growth:
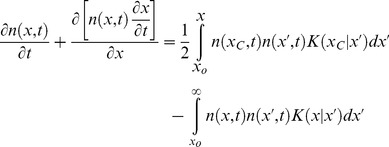
(9)


The density function 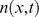 is defined such that 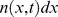 is the fraction of aggregates with sizes between 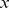 and 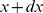 at time 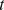 per unit volume of the culture. The size 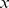 corresponds either to the aggregate volume or mass since the buoyant density of cells does not vary significantly during the cell cycle [21], [22]. The mass of individual cells was taken as 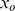 and 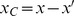. Aggregate breakage was not observed particularly after the first day of culture and therefore was not considered.

The rate of aggregate size change, 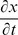, due to cell proliferation was modeled after the Gompertz equation [23]:
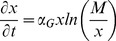
(10)where *M* is the aggregate mass reached as 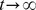 and 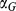 is a constant characteristic of the cell proliferation. Both parameters were estimated from temporal data of aggregates in static cultures.

Different aggregation kernels 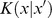 have been reported in various systems [24], [25]. Given the non-rigid nature of cells/aggregates, we applied here an aggregation kernel (Eq. 11) for liquid-liquid dispersions assuming dynamic deformation of the colliding bodies [26]. The coalescence efficiency (exponential term) decreases with increasing average size of the colliding aggregates. The parameters 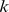, 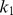 and 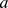 are obtained from ESC aggregate size distributions measured in spinner flask cultures.
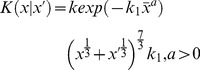
(11)


The PBE was expressed with respect to the aggregate radius, which was measured experimentally, assuming spherical aggregates. However, the density function was transformed in terms of the logarithm of the aggregate radius normalized to that of a single cell (∼7.5 µm) to allow for an extended range of aggregate sizes to be considered (see [17] and *Materials S1*).

The integro-partial differential PBE was solved numerically by finite (backward) differences over equally spaced nodes in the *lnr* domain. Kernel parameters for different agitation rates were obtained from aggregate size data. The total number of aggregates and biomass were calculated as the zeroth and first moments of the cell distribution.

#### Integration of PBE and reaction-diffusion models

The PBE and reaction-diffusion models were combined for predicting the O_2_ profile in stirred-suspension cultures of ESC aggregates (**Fig. 1**). The total number of aggregates 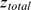, size distribution of aggregates 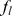, oxygen concentration in the medium bulk 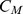, working volume of bioreactor 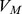 (assumed equal to that of the medium) and agitation rate 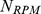 were used for initialization of the integrated model (see *Materials S1*). Cell growth and aggregation were modeled (Eq. 10–11) and the consumption of O_2_ by all the aggregates in the bioreactor was determined (Eq. 7). This required knowledge of the O_2_ concentration distribution within individual aggregates (Eq. 1). Subsequently, the bulk O_2_ level 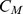 was updated based on the net effect of O_2_ consumption and supply:
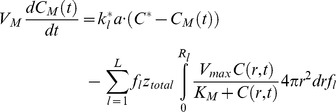
(12)


The rate of O_2_ supply through headspace aeration was:
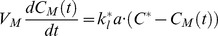
(13)where 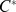 is the O_2_ partial pressure in the gas phase (95% air/5% CO_2_, 37°C, 760 mm Hg), 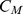 is the oxygen partial pressure in bulk medium, 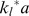 is the mass transfer coefficient at the gas-liquid interface. Values of 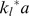 were determined experimentally (see *Materials S1*).

## Results

### Porosity and tortuosity of ESC aggregates

The transport of O_2_, nutrients and pluripotency-maintaining or differentiation-inducing factors to and within stem cell aggregates is greatly influenced by the aggregate ultrastructure. Moreover, whether the intra-aggregate ultrastructure is strongly affected by culture conditions in SSBs -particularly agitation- remains unknown. Thus, before proceeding with the solution of the diffusion-reaction model for ESC aggregates, we used CLSM and image analysis to determine two ultrastructural descriptors –porosity (ε) and tortuosity (τ)– for mESC and hESC aggregates formed in dishes or spinner flasks at different stirring rates (**Fig. 1A**). The porosity of mESC aggregates in spinner flasks at 100 rpm was 0.229±0.016 at 24 hours (**Fig. 1B**) and decreased slightly after 3 (0.218±0.011) and 5 days (0.220±0.011) of culture suggesting that aggregates were undergoing compaction. For 3-day cultures the ε values for 60 and 80 rpm were 0.217±0.02 and 0.210±0.025, respectively (**Fig. 1C**). When cultured in dishes, mESCs formed looser aggregates with ε = 0.259±0.041 (day 3). Yet, no statistically significant differences were noted in the porosity between aggregates cultured in dishes and spinner flasks at different agitation rates. Human ESCs in static culture formed aggregates with a porosity of 0.254±0.017 (**Fig. 1D**) whereas hESC aggregates from spinner flasks at 45 and 60 rpm displayed porosities of 0.258±0.011 and 0.270±0.007, respectively.

The tortuosity of mESC aggregates at 100 rpm was 1.427±0.083 at 24 hours and did not change significantly after 3 (1.504±0.093) and 5 days (1.547±0.181) of culture (**Fig. 1B**). Moreover, clusters formed at 60 rpm (1.460±0.035), 80 rpm (1.534±0.086) and in dishes (1.457±0.422) exhibited similar tortuosity. Human ESC aggregates were characterized by slightly higher average τ values than mESC clusters (**Fig. 1D**). After 4 days in stirred suspension an average τ of 1.612±0.135 (45 rpm) or 1.551±0.086 (60 rpm) was determined whereas the tortuosity of dish aggregates was 1.638±0.136.

Our findings provide a first account of porosity and tortuosity values determined directly for mESC and hESC aggregates formed in dish and stirred-suspension cultures at different times and under typical agitation rates. The data did not show strong dependence of the ultrastructural descriptors considered here on the cultivation mode, i.e. dish vs. stirred suspension nor on the stirring speeds tested.

### Effective diffusion and consumption of O_2_

**Figure 2:**
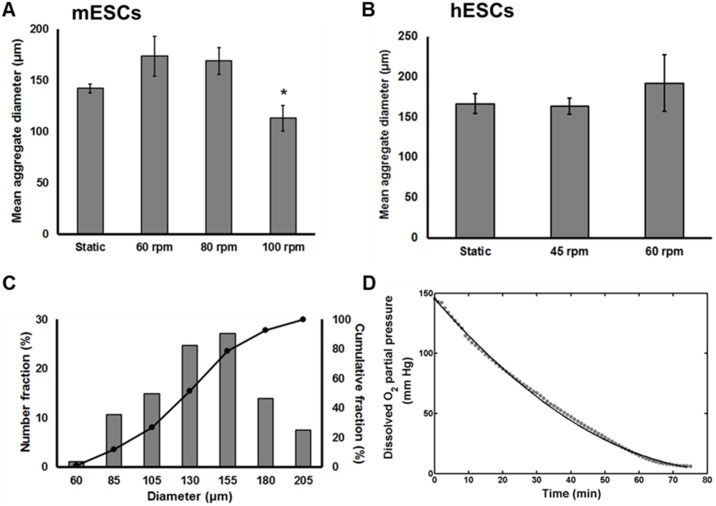
Experimental measurement of ESC aggregate size and O_2_ consumption profiles for static and spinner flask cultures. Mean aggregate diameter data are shown for dish and bioreactor cultures at different agitation rates for (A) mESCs (*p<0.05 vs. 60 and 80 rpm) and (B) hESCs. (C) A representative ESC aggregate size distribution is shown (mESC culture at 100 rpm). (D) Experimental (solid line) and simulation (dotted line) results for the consumption of O_2_ over time. Parameter (V_max,_ D, and K_M_) values were acquired by fitting the transient reaction-diffusion model to the experimental data for different agitation rates (Table 1).

A transient reaction-diffusion model was employed to determine the values of the diffusion coefficient 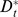 and consumption (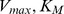) of O_2_ in the ESC aggregates from experimentally measured O_2_ concentration values in the medium. First, the mean size was determined of aggregates in dishes and stirred-suspension cultures at different agitation rates (**Fig. 2A**). At the end of the culture, mESC aggregates at 100 rpm were smaller (mean diameter: 113.04±15.64 µm, p<0.05) than those at 60 rpm (173.8±23.9 µm) and 80 rpm (169.01±16.13 µm). The mean diameter of hESC aggregates at 45 and 60 rpm at day 4 was 163.8 and 192.45 µm, respectively and similar to those in static cultures (166.61±12.4 µm) (**Fig. 2B**). For each sample, the number fraction 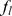 distribution was determined (**Fig. 2C**).

Cells within aggregates consumed O_2_ resulting in a gradual decrease of the DO level in the medium (**Fig. 2D**). Typically, DO measurements were performed until the O_2_ concentration in the medium dropped below 10 mm Hg. Solution of the model coupled with experimental data on DO, yielded values for 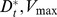 and 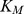 (**Table 1**). It should be noted that parameter values were calculated for a minimum (critical) O_2_ concentration at the aggregate center of either (i) 0 mm Hg, or (ii) 8 mm Hg [27]. Irrespective of the minimum O_2_ level considered, the values of 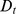 (see Eq. 2) were lower than that of the diffusion coefficient 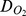 of O_2_ in pure water at 37°C (2.78×10^−5 ^cm^2^/s). As a measure of maximum O_2_ consumption rate, V_max_ was higher for mESCs in suspension culture at 60 rpm than for hESCs (p<0.05). Mouse ESCs also displayed greater V_max_ at 100 vs. 60 rpm suggesting an effect of the agitation on O_2_ metabolism (p<0.05). The mean V_max_ for hESCs at 45 and 60 rpm did not differ significantly.

**Table 1:** Parameter values with a critical concentration of 8 mm Hg.

Culture condition	D_t_ (×10^5 ^cm^2^/s)	V_max_ (×10^8^ mol/cm^3.^s)	K_M_ (×10^8^ mol/cm^3^)
mESCs			
**Static culture (aggregates)**	1.631±0.19 [0.242±0.024]	2.615±0.997 [4.732±0.318]	2.81±1.904 [0.518±0.165]
**60 rpm**	1.716±0.223 [0.364±0.02]	2.667±0.378*,# [5.055±0.163]	2.721±0.385 [0.782±0.329]
**80 rpm**	1.283±0.864 [0.418±0.05]	3.103±1.03 [3.602±1.06]	4.575±1.915 [0.392±0.233]
**100 rpm**	2.375±0.199 [0.386±0.114]	4.831±0.593 [7.322±0.83]	4.063±0.693 [0.563±0.06]
**hESCs**			
**Static culture (aggregates)**	1.20±0.170 [0.217±0.022]	0.995±0.464 [2.029±0.478]	3.473±0.232 [0.062±0.033]
**45 rpm**	1.536±0.322 [0.547±0.093]	1.16±0.355 [1.591±0.963]	3.23±0.257 [1.564±0.155]
**60 rpm**	1.163±0.454 [0.69±0.223]	1.36±0.427 [1.266±0.542]	4.126±1.138 [0.876±0.443]

Values in brackets are calculated with a 0 mm Hg critical concentration.

*p = 0.005 [0.019] for V_max_ at 60 vs. 100 rpm,

# p = 0.032 [0.001] for V_max_ for mESCs vs. hESCs at 60 rpm.

### Oxygen profile within ESC aggregates in static and stirred-suspension cultures

**Figure 3:**
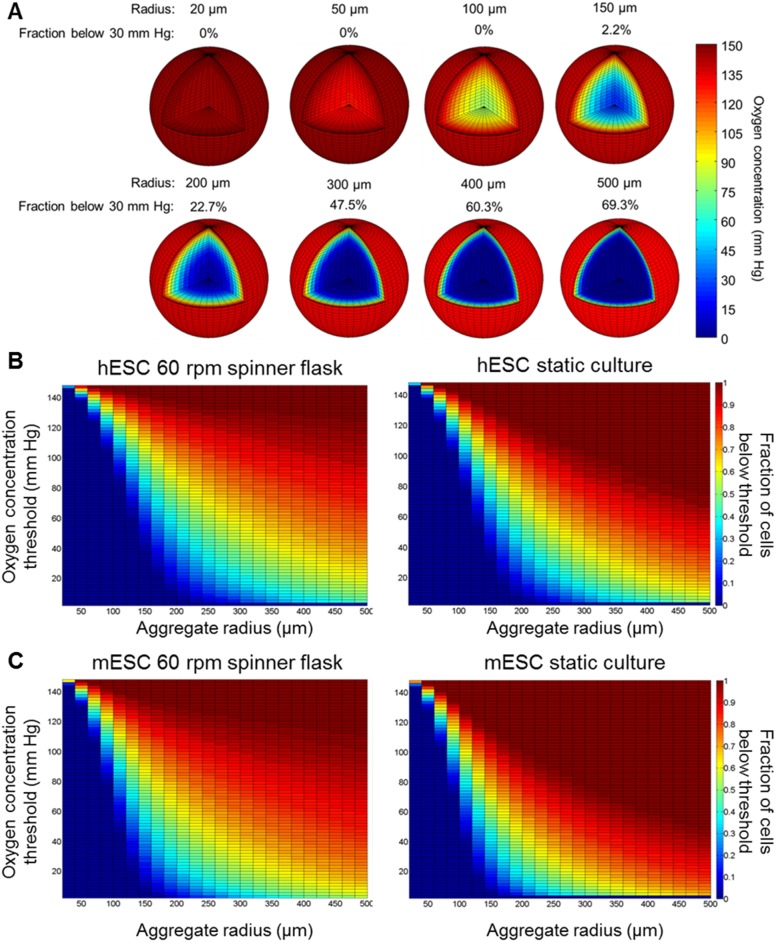
Steady-state O_2_ profile within ESC aggregates of varying sizes. (A) Three-dimensional distribution of O_2_ and hypoxic cell fraction of hESC aggregates with radius ranging from 20 µm to 500 µm. The bulk oxygen level was taken as 148 mm Hg. Fractions of ESCs exposed to O_2_ below a particular threshold (y-axis) for different size aggregates cultured at 60 rpm or in dishes (static) shown for (B) hESCs and (C) mESCs. Results were obtained assuming that all cultured aggregates were the same size. The minimum intra-aggregate O_2_ level was fixed at 8 mm Hg (critical concentration).

Once the values of 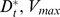 and 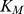 were determined, the reaction-diffusion model was implemented to calculate the O_2_ profile for ESC aggregates of different size. The diffusion of O_2_ becomes limited as the aggregate radius increases leading to reduced O_2_ levels for cells residing closer to the core. To better illustrate this, the O_2_ distribution was calculated in hESC aggregates sampled from spinner flask cultures assuming a constant O_2_ concentration in the bulk of 148 mm Hg (saturation) (**Fig. 3A**). Because hypoxia studies typically employ pO_2_ of ∼30 mm Hg (4–5% O_2_) [5], we calculated the fractions of ESCs exposed to O_2_ below this level for different aggregate sizes. Human ESC aggregates smaller than 200 µm were normoxic throughout their volume. In contrast, almost 23% of the hESCs in aggregates larger than 400 µm were under hypoxia. This fraction increased to 70% for aggregates with 1,000 µm diameter (**Fig. 3A**). The O_2_ profiles in hESC aggregates of various sizes maintained in suspension (60 rpm) or static culture are shown in **Fig. 3B**. Similarly, the O_2_ distributions within mESC aggregates cultured at 60 rpm or statically are also shown (**Fig. 3C**). It should be noted that the results shown in **Figs. 3B–C** were obtained assuming that all aggregates in culture are the same size. Simulation results for additional agitation rates are shown in **Figure S1**.

In general, the fractions of cells experiencing an O_2_ concentration below a particular threshold were lower in stirred suspension compared to dish cultures for given size clusters. For instance, the hypoxic cell fractions are larger in the plots from dishes compared to spinner flask cultures for the same size aggregates (**Figs. 3B–C**). This is most likely due to the faster transfer (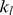) of O_2_ from the medium bulk to each aggregate in agitated vessels compared to static cultures (Eq. S2). Practically however, we rarely observed aggregates with a radius over 300 µm and only in static cultures. The higher hindrance for O_2_ (and nutrient) transport to large clusters possibly impacts ESC viability and reduces proliferation by stimulating differentiation. This and the agitation-induced shear in spinner flask cultures may limit further growth of bigger aggregates.

Aggregates from stirred-suspension cultures showed similar O_2_ profiles across all the agitation rates we tested (**Fig. S1**). As the aggregate size increased, cells positioned over 100 µm from the aggregate surface experienced less than 10 mm Hg of O_2_. Compared to hESCs, the fractions of mESCs under the same O_2_ concentration threshold were greater for equal size aggregates owing most likely to the higher V_max_ of the latter. The difference was most significant for aggregates with a radius between 100–250 µm.

Nevertheless, the expansion of mESCs and hESCs in spinner flasks gave rise to aggregate diameters mostly below 200–300 µm (**Fig. 2**). Thus, if a normoxic concentration of O_2_ can be maintained in the bulk only 2.2% of the cells (located in the aggregate center) experience O_2_ tension below 30 mm Hg (**Fig. 3A**). In fact, when we analyzed cells by qPCR for HIF1α expression (**Fig. S2**) there was no notable differences between static and stirred-suspension cultures. Also, compared to monolayer cultures at normoxia, HIF1α expression increased only slightly for mESCs (<2.0-fold) and hESCs (<1.5-fold) within aggregates.

### PBE model for ESC aggregation in stirred-suspension culture

For the O_2_ profiles presented above, the O_2_ concentration in the medium was assumed to be fixed at saturation (∼148 mm Hg). This assumption is valid if O_2_ can be supplied efficiently and continuously to the cultivation system or the culture is sparse so that O_2_ consumption is trivial. However, stirred-suspension cultures accommodate high cell densities resulting in fluctuating O_2_ levels in the bioreactor. Agitation and proliferation also drive the formation and growth of aggregates over time with concomitant changes in the intra- and inter-aggregate concentrations of O_2_. Given the multitude of effects of O_2_ on stem cell physiology, predicting the time-variant distribution of O_2_ in scalable ESC aggregate cultures is highly desirable.

**Figure 4:**
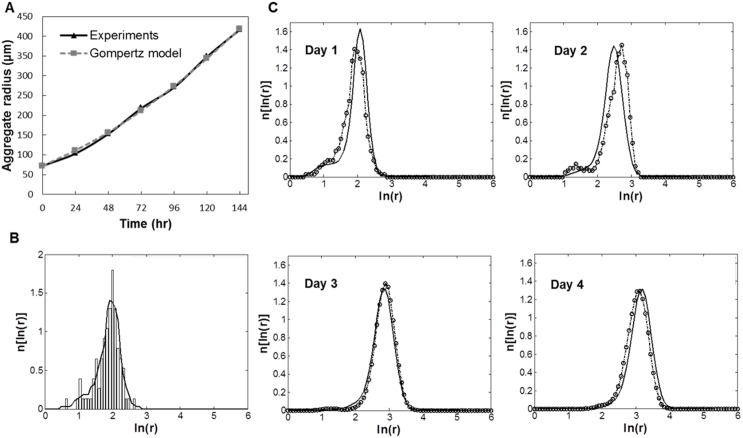
PBE model for the ESC aggregate culture in spinner flasks. (A) Mouse ESC aggregates in dishes were monitored and their size was measured over several days. The experimental data for aggregate growth due to mESC proliferation were compared with the Gompertz model. Relevant parameters were determined: M = 9.71×10^6^, α_G_ = 5.72×10^−3 ^hr^−1^. (B) Representative data of size (radius) distribution for mESC aggregates cultured at 60 rpm (day 1). Histogram and a continuous distribution curve are shown. (C) Model size distributions (lines) shown for a 96-hour culture at 60 rpm. The original distributions (dash-circle lines) for each time point are shown for comparison.

To that end, a PBE model was developed for the temporal evolution of the ESC cluster size distribution in an SSB. The growth rate of ESC aggregates due to cell proliferation was described by the Gompertz equation. For this purpose, the growth of sparsely cultured mESC aggregates in dishes was monitored and the Gompertz parameters were evaluated (M = 9.71×10^6^, α_G_ = 5.72×10^−3 ^hr^−1^) based on the recorded size changes (**Fig. 4A**). The Gompertz model accurately described the growth of mESC aggregates (R^2^ = 0.99, n = 20). The maximum attainable size for mESC aggregates was calculated at 1600 µm (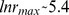).

Parameters for the aggregation kernel were determined from size data of mESC aggregates cultured in spinner flasks (**Fig. 4B**). It should be noted that the kernel parameters were calculated from data of cell cultures at 0–72 hours (**Table 2**). At longer times, aggregation was negligible and cell proliferation drove the increase in cluster size. With increasing agitation rate, the parameter value trends are such that the coalescence frequencies (i.e. collisions leading to aggregate formation) become lower. At 60 rpm, the mean aggregate radius increased to ∼105 µm on day 2 (mainly due to collision-driven aggregation) in line with the model predictions (**Fig. 4C**). Subsequent increase in aggregate size was less pronounced as the mean radius was approximately 150 µm (day 4). The corresponding average size at higher agitation rates was lower (e.g. ∼90 µm at 100 rpm, **Fig. S3**). The simulation results were corroborated by observations that a lower agitation rate generally promotes formation of larger aggregates. It should also be noted that the cell viability was not affected significantly by agitation (>90%).

**Table 2:** Kernel parameter values at different agitation rates.

Agitation rate (rpm)	*k* (hr^−1 ^µm^−7/3^)	*k_1_* (µm^−3*α*^)	*α* (unitless)
**60**	1.26±0.77×10^−3^ *	1.94±1.12×10^−4^	8.06±0.79×10^−1^ *
**80**	6.36±4.47×10^−6^	4.62±3.25×10^−4^	5.96±0.085×10^−1^ *
**100**	6.53±6.23×10^−6^	4.11±0.63×10^−5^	9.91±0.65×10^−1^ *

The parameter values were shown as mean ± st.dev. calculated by model simulation to experimental data from mESC cultures (at least 3 independent runs per agitation rate).

*p<0.05 when compared to the mean values obtained for the other agitation rates.

Taken together, the PBE model developed here allowed the prediction of the temporal evolution of the ESC aggregate size distribution (and the total aggregate number and biomass; see below). Simulation results were in good agreement with the experimental observations from stirred-suspension ESC culture at different agitation rates.

### Prediction of O_2_ profile and hypoxia fraction in ESC aggregate stirred-suspension culture

**Figure 5:**
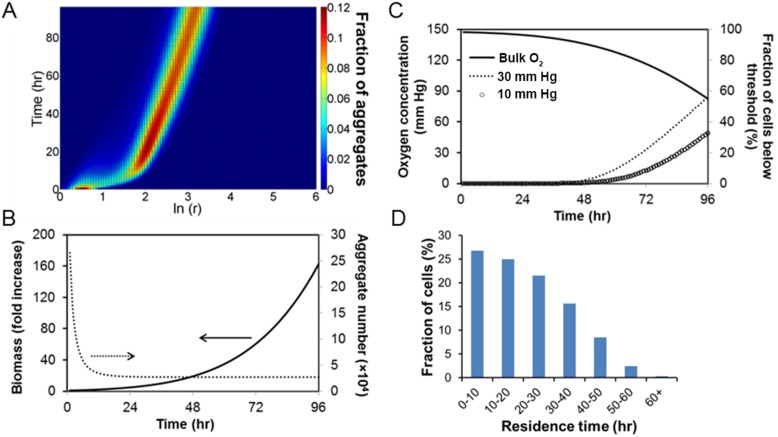
Model prediction of cluster size and O_2_ concentration distribution in a stirred suspension bioreactor of cultured mESC aggregates. (A) Density plot of mESC aggregate size distribution during a 4-day culture. (B) Total aggregate number and biomass of mESC aggregates over 4 days of culture. (C) Oxygen concentration in the medium bulk and fraction of ESCs experiencing pO_2_ below 30 mm Hg. The model was run for a 50-ml working volume and 60 rpm with mESCs seeded at 5×10^4^ cells/ml. Specific model parameters are from Tables 1–2. (D) Distribution of residence time of ESCs exposed to ≤30 mm Hg pO_2_ during a 4-day culture.

With the PBE model of ESC aggregation in the SSB in place, the O_2_ distribution in the medium and within ESC aggregates was computed by coupling the diffusion reaction and PBE models. For this purpose, the coefficient 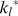 was measured for the O_2_ transfer via headspace aeration to the bulk medium (air-liquid interface, **Fig. S4**). The slope (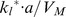) was 4.87±0.13 hr^−1^ yielding a 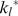 of 16.57±0.43 cm/hr for our cultivation system. Simulations were run for spinner flask cultures of mESCs at 60 rpm and a seeding density of 5×10^4^ cells/ml. The temporal size distribution of mESC aggregates is shown in **Fig. 5A**. During the first day, there was a significant increase in mean cluster size mainly due to aggregation. By day 3, mESC aggregates had a mean diameter of around 200 µm in agreement with our cell culture data. The total number of aggregates dropped substantially during the first day due to aggregation but remained relatively steady afterwards (**Fig. 5B**). At day 4, the calculated total number of aggregates was around 2.7×10^4^/50 ml of culture similar to our experiments and the corresponding biomass had increased 160-fold.

The fractions of cells exposed to various levels of O_2_ in spinner flask cultures were also predicted. Here, the fractions were computed of cells experiencing O_2_ tension below 30 mm Hg, which may be favorable for maintenance of ESC pluripotency (**Fig. 5C**). We also calculated the fractions for extremely low pO_2_ (<10 mm Hg), which hampers ESC self-renewal and induces death [5]. The pO_2_ in the medium decreased to 82 mm Hg after 4 days with 58% of cells experiencing O_2_ less than 30 mm Hg. The corresponding fraction of ESCs at 10 mm Hg or less was 32%. If the O_2_ concentration in the culture bulk was fixed at 148 mm Hg throughout the cultivation period, only 33% of cells would be at pO_2_<30 mm Hg by day 4 (data not shown).

In addition, the residence time distribution (**Fig. 5D**) shows that mESCs entered low O_2_ level (<30 mm Hg) at different time points. This variation is attributed to the distributed size of cell clusters and random aggregation under stirring. The data clearly illustrate that although a group of cells is exposed to a particular O_2_ range (e.g. below 30 mm Hg), not all cells within the group experience the O_2_ level for the same amount of time. Therefore, information about the fraction of cells at different pO_2_ levels should be coupled to residence times provided by the model presented here. Our findings show that stem cell aggregate size heterogeneity is linked to population heterogeneity with respect to the duration of exposure at different levels of O_2_ concentration despite the fairly homogeneous environment induced by mixing in SSBs.

## Discussion

The transport of factors among cells within 3D structures such as aggregates is an important aspect of the performance of cultivation systems. The importance becomes even more pronounced for stem cell culture modalities given the central role of O_2_ in pluripotency and lineage specification. We employed a transient reaction-diffusion model to predict the O_2_ profile inside ESC aggregates of varying sizes. The effective diffusivity and consumption rate parameters of O_2_ in hESC and mESC aggregates were determined for the first time based on experiments without a steady state constraint. A PBE model was further developed for the SSB culture of ESC aggregates. The combined diffusion-reaction/PBE framework was utilized to predict the aggregation outcome and the O_2_ profile inside ESC clusters and the medium bulk in stirred-suspension over time. As a result, the fractions of cells exposed to particular levels of O_2_ and pertinent residence times were determined allowing to draw conclusions about the ensuing population heterogeneity and state of the cells (e.g. viability, differentiation potential).

The oxygen level to which ESCs are exposed to significantly affects cell fate decisions. Hypoxia (∼30 mm Hg) does not modulate adversely the proliferation rate of hESCs, which exhibit reduced spontaneous differentiation and chromosomal abnormalities [5], [6], [28]. Human ESCs and iPSCs exhibit differentiation efficiencies along particular lineages dependent on O_2_ availability. For instance, low pO_2_ enhances hESC differentiation to chondrogenic, endothelial and cardiac cells [7], [8], [29]. Mouse ESC EBs generate significantly more myeloid and erythroid progenitors when cultured at 3% O_2_ instead of 21% O_2_ [30]. However, extremely low pO_2_ can be detrimental for cells. Thus, delicate pO_2_ control is essential in culture systems to avoid hampering stem cell growth, viability and self-renewal or commitment. This is particularly relevant in scaling up stem cell cultivation where local concentration gradients and transfer of factors are more pronounced. The model presented in this study permits the prediction of the actual O_2_ level facilitating informed decisions about dynamic pO_2_ control and optimal aggregate size in systems for stem cell expansion.

For the results reported here, an implicit assumption was made that the O_2_ consumption rate (i.e. K_M_ and V_max_) does not vary with time or the location of cells within each cluster. Although this is a premise that is common with previous reports, we cannot exclude the possibility of ESC metabolism adaptation to available O_2_ levels. For example, V_max_ was estimated to vary with pO_2_ albeit within the same order of magnitude [31]. Human ESCs reportedly lower their O_2_ uptake rate when the dissolved O_2_ decreases from 21% to at 5% and 1% O_2_ [32]. Such adjustment may aid the cells to maintain the concentration of available O_2_ above zero at the core of aggregates with a diameter >200 µm thereby reducing the likelihood for necrosis (**Fig. S1** in the absence of the constraint of 8 mm Hg critical pO_2_). However, further studies are warranted on the adaptation of O_2_ consumption by pluripotent stem cells in response to microenvironment O_2_ levels. With more information becoming available, our model can accommodate spatially (e.g. radial distance from the aggregate surface) and temporally changing O_2_ consumption rates.

Our study also raises the issue of ‘residence time’ (duration of exposure) of cells especially to low O_2_ levels. To our knowledge, this has not been investigated previously and even in reports of stem cell aggregate cultivation in stirred-suspension vessels, emphasis is placed on the actual pO_2_ the cells experience. It is important to note that even after the first 5 minutes of mESC culture in 2.2% O_2_, increases are observed in HIF-1α expression and reactive O_2_ species [33]. At 12–24 hours of hypoxia, cell-cycle regulatory proteins such as cyclins D1 and E, CDK2 and CDK4 along with retinoblastoma phosphorylation increase yielding a larger fraction of mESCs in the S phase and higher overall cell numbers. Accelerated cell proliferation in aggregate regions of hypoxia will lead to increased O_2_ consumption further depressing pO_2_ levels. As our model indicates, an increasing fraction of cells will reside in the hypoxic region of the aggregates which grow continuously. The ‘cut-off’ time of cell exposure to low pO_2_ before hypoxia-induced changes (e.g. cell differentiation, apoptosis etc.) become irreversible is unknown. Such information will be essential in formulating strategies (e.g. by modulating agitation (**Table 2**)) to achieve ESC aggregate sizes so that a high cell fraction remains pluripotent for downstream differentiation to a desired phenotype.

The effects of O_2_ on gene expression are mediated largely by HIF transcription factors and as noted, mESCs upregulate HIF-1α within 5 minutes of exposure to 2.2% O_2_. Cells in our cultures did not exhibit significant differences in HIF-1α expression under different agitation rates (**Fig. S2**). This may be attributed to the average cluster diameter, which generally ranged between 200–300 µm (**Fig. 3**), leading to rather limited fractions of cells exposed to 2.2% O_2_ or less. Yet, we acknowledge that HIF-1α transcripts (probed by qPCR) may not be representative of the corresponding protein amount and activity levels. Moreover, the qPCR results are population ‘averages’ and HIF expression variation at locales with different pO_2_ cannot be discerned. Lastly, changes due to fluctuating O_2_ can be brought about by HIF-independent mechanisms such as the environmental sensing mammalian target of rapamycin (mTOR) [34].

Although this study focused on the transport of O_2_ in ESC aggregates in dish and stirred-suspension cultures, the concurrent availability and consumption of other components such as nutrients and factors for self-renewal or differentiation should be considered. A steady state analysis of the diffusion of glucose and (generic) cytokines (in addition to O_2_) in human EBs was recently presented [12]. The diffusivities of glucose and cytokine were lower than that of O_2_ suggesting that their transport places further limits on ESC aggregates to avoid cell starvation and death. Such limitations on transport may actually hinder the growth of aggregates and cause their size to stabilize. Interestingly enough, Cameron et al. [35] reported that over 21 days hESC aggregates displayed a maximum size at day 10 ranging between 400 and 500 µm without further increase. These observations support the choice of the Gompertz equation for modeling aggregate growth due to cell proliferation. The Gompertz equation in our study suggested a maximum radius larger than our experimental observations. This discrepancy may be explained by the fact that experimental data were obtained from static cultures with relatively constant bulk concentration of substrates (oxygen, glucose, etc.) due to daily medium changes whereas realistically these concentrations should decrease markedly over time. The maximum radius attained by ESC aggregates may be lower if substrate-dependent growth kinetics are considered. Additional mass balance equations for nutrients and growth factors can be incorporated in the model presented here to provide a more accurate representation of the bioreactor stem cell culture.

The diffusion and consumption of O_2_ in mESC and hESC cultures has been investigated in various studies [12], [13], [31], [36], [37] although only in a subset of these reports parameters were determined from experiments. The values reported for V_max_ and K_M_ are the same order of magnitude (10^−8^ mol.cm^−3^.s^−1^ and 10^−8^ mol.cm^−3^, respectively) as those presented here. The O_2_ consumption rate for mESCs was previously [38] measured at 4×10^−17^ (mol/cell.s), i.e. close to our observed rate assuming cells are spherical with a 15 µm diameter. Values for the diffusion coefficient based on experimentally determined ultrastructural ESC characteristics of aggregates such as porosity and tortuosity were not available to date in the literature for comparison. In fact, the ε and τ values did not vary significantly with the culture modality, i.e. dish or stirred suspension and the agitation speeds tested. This could suggest that cells adopt particular arrangements in the aggregate interior without significant influence from external mechanotransduction but this warrants further investigation. Such organizations may be mediated by cytoskeletal actions, which are central to the assembly of other cell types into aggregates [39].

It should be noted that no overt changes were observed in the values of V_max_, K_M_ or D_t_ at different agitation rates. In part this can be explained by the fact that the majority of cells are within aggregates not experiencing the different levels of shear induced by the stirring rates tested here. Moreover, the DO measurements were performed in PBS-based solution with a known Bunsen solubility coefficient. This facilitates the comparison with findings from previous studies and eliminates potential discrepancies due to differences in culture media utilized in those reports. Even though we cannot rule out potential changes in parameter values if the measurements were performed in culture medium, these changes are expected to be minimal as shown for other systems [36], [37], [40].

A multiscale approach was described here with the coupling of the transient diffusion-reaction model for individual clusters with the PBE for the temporal evolution of size for ESC aggregate population in the bioreactor due to cell proliferation and collisions. The kernel used in this study comprises two parts: (i) The sum of cubic roots of volumes of the aggregates colliding raised to the power of 7/3 is applicable for a shear field with non-linear velocity profile [25], [26] as in the case of bioreactors with impellers and baffles. This is in contrast to the Smoluchowski kernel [41] used for cell aggregation (e.g. of platelets [42]) assuming a linear shear flow. (ii) A multiplicative factor dependent on operating conditions including the agitation rate represents the coalescence frequency written as the product of the collision frequency and the coalescence efficiency. Use of the kernel assumes that the aggregates are not rigid spheres but deform during collision. More importantly, the coalescence efficiency (collisions leading to new aggregate formation) decreases with increasing aggregate size in line with our experiments. Because of these attributes, the kernel is deemed suitable for describing the aggregation of stem cells in stirred-suspension vessels despite its original derivation for droplet coalescence in liquid-liquid dispersions. Explicit functions of the kernel parameters dependent on agitation rates can be estimated from the data (**Table 2**), and additional operating variables may be included (e.g. cell seeding density which was kept constant for all experiments here). Since transient size distributions of ESC aggregates cultivated in spinner flasks are available, a proper approach to determining the kernel for the PBE model is to solve the inverse problem [43]. Work is underway for deriving kernel functions via solving the inverse problem and comparing the solution to the functional utilized in this study.

The approach described here is certainly not without limitations but amenable to improvements. As already mentioned, the transport and consumption or secretion of factors other than O_2_ were not considered. Fluctuations in the consumption of nutrients and/or accumulation of waste will almost certainly affect the microenvironment within aggregates. These can be considered by expanding the model, for instance, to include reaction-diffusion equations (see Equation 1) for additional species. Moreover, O_2_ consumption was assumed invariant with the position of each cell within aggregates. Pertinent variations can be introduced by considering different expressions for V_max_ and K_M_ based on spatial criteria or other conditions (e.g. concentration of particular nutrients). In the context of envisioned stem cell process designs, the model in its current or expanded form can be used in conjunction with investigating relevant bounds in culture variables.

Nonetheless, the framework presented here can be applied widely to address both basic questions of stem cell physiology and issues surrounding relevant bioprocesses utilizing SSBs. The model can be employed to generate maps of concentrations of O_2_ (and other factors) expediting research on the role of hypoxia on stem cell self-renewal or commitment along particular lineages. Gaining a better understanding of the effects of O_2_ on fate selection is critical in developing optimal strategies for stem cell differentiation. The design and control of stem cell bioprocesses will also benefit from quantitative multiscale approaches such as the one described here, thereby bringing us closer to the realization of the potential of stem cell therapeutics.

## Supporting Information

Figure S1
**Steady-state O_2_ profile for ESC aggregates of different sizes cultured under different conditions. Oxygen concentration profile for (A) hESC aggregates cultured in static or stirred suspension (45 rpm and 60 rpm) culture, and (B) mESC aggregates cultured in static or stirred suspension (60 rpm, 80 rpm and 100 rpm) culture.** The distance in the horizontal axis is measured from the aggregate center. The O_2_ level in the medium bulk was kept fixed at 148 mm Hg for all conditions. The aggregate radius ranged from 30 µm to 500 µm. Values for V_max_, K_M_ and 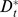 were taken from Table 1.(TIF)

Figure S2 **HIF1α gene expression in (A) mESCs and (B) hESCs cultured as aggregates in dishes and spinner flasks.**
(TIF)

Figure S3 **Comparison of experimental data (dash-circle lines) and model (solid lines) results for different agitation rates at day 4 of culture: (A) 60 rpm, (B) 80 rpm, (C) 100 rpm.**
(TIF)

Figure S4 **Measurement of O_2_ transfer to the medium through headspace aeration in spinner flasks.** (A) Oxygen concentration during the experiment. The dissolved O_2_ in the medium bulk was first depleted by gassing with nitrogen. After the depletion phase, air was allowed to fill the space above the medium and the O_2_ level was measured. (B) Linear regression of the recovery phase for O_2_ marked in (A). The standard deviation was calculated from at least three independent experiments. The O_2_ transfer coefficient was determined from the slope of the curve.
(TIF)

Materials S1 **Supplemental information on cell culture, image analysis, modeling methods and measurements of bioreactor O_2_ transfer coefficient and HIF1a gene expression levels.**
(DOCX)
