# Supplementary figures and images for: Oxygen Transport and Stem Cell Aggregation in Stirred-Suspension Bioreactor Cultures
Source: PLoS One. 2014 Jul 17;9(7):e102486. doi: 10.1371/journal.pone.0102486 (PMC4102498; doi:10.1371/journal.pone.0102486)

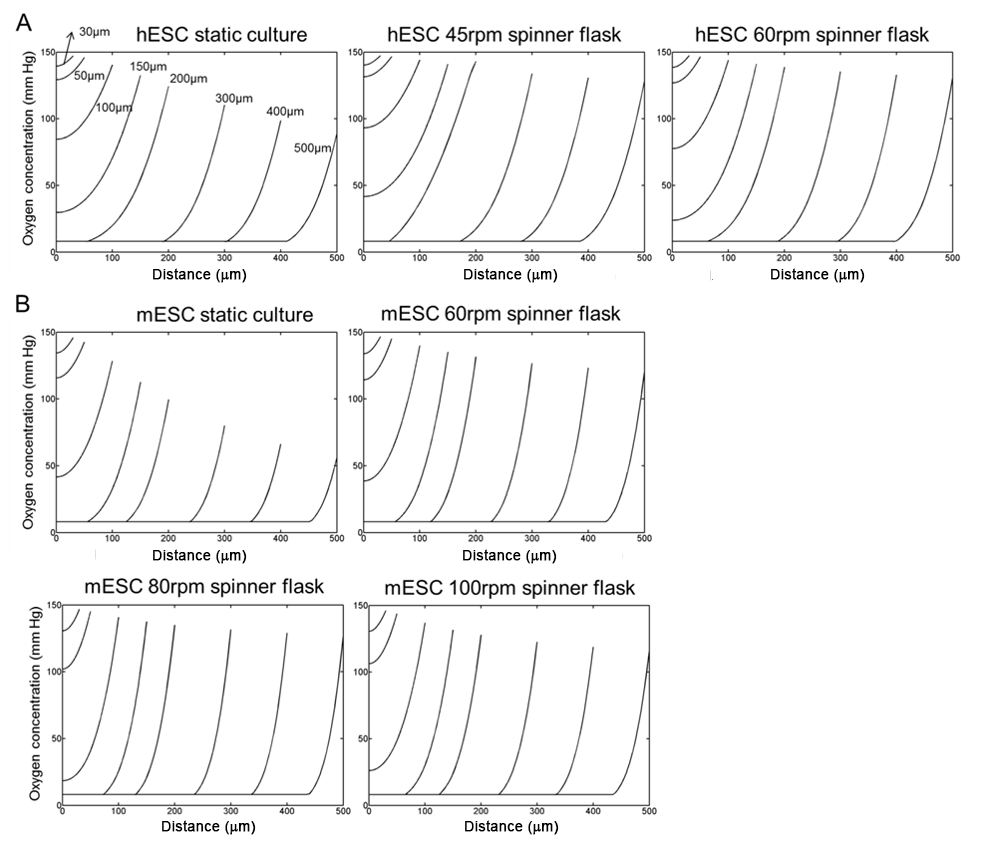

Supplement: Figure S1 — Steady-state O2 profile for ESC aggregates of different sizes cultured under different conditions. Oxygen concentration profile for (A) hESC aggregates cultured in static or stirred suspension (45 rpm and 60 rpm) culture, and (B) mESC aggregates cultured in static or stirred suspension (60 rpm, 80 rpm and 100 rpm) culture. The distance in the horizontal axis is measured from the aggregate center. The O2 level in the medium bulk was kept fixed at 148 mm Hg for all conditions. The aggregate radius ranged from 30 µm to 500 µm. Values for Vmax, KM and were taken from Table 1. (TIF) [file pone.0102486.s001.tif]

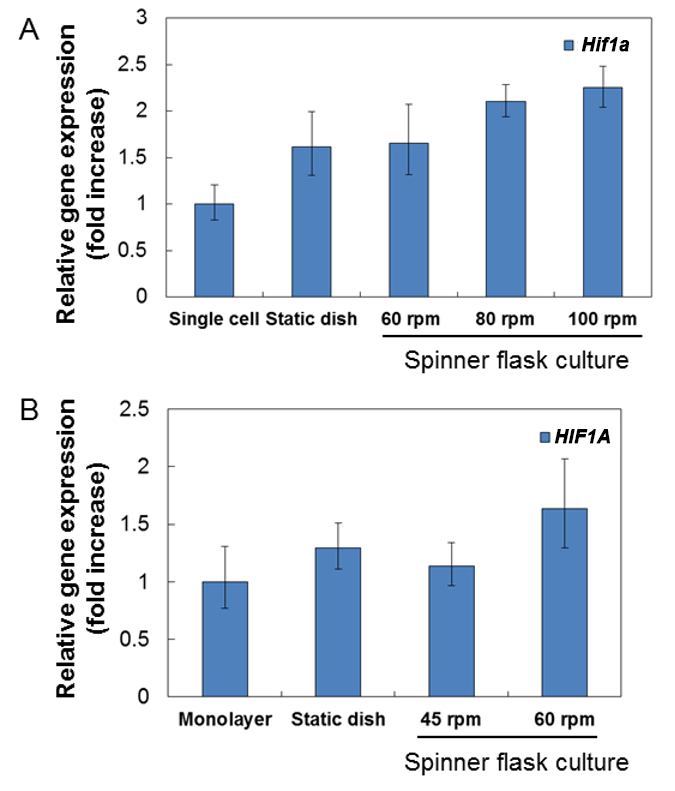

Supplement: Figure S2 — HIF1α gene expression in (A) mESCs and (B) hESCs cultured as aggregates in dishes and spinner flasks. (TIF) [file pone.0102486.s002.tif]

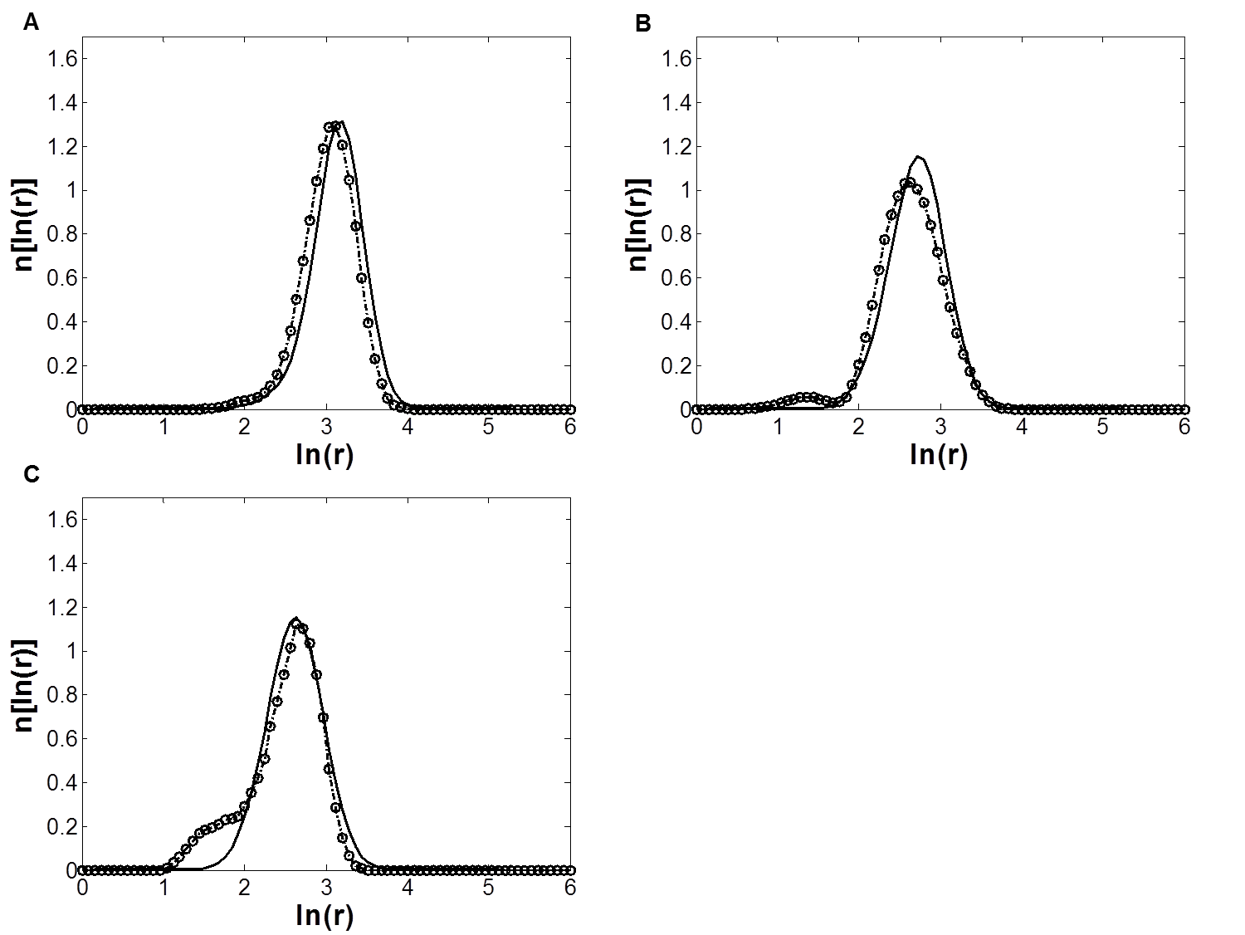

Supplement: Figure S3 — Comparison of experimental data (dash-circle lines) and model (solid lines) results for different agitation rates at day 4 of culture: (A) 60 rpm, (B) 80 rpm, (C) 100 rpm. (TIF) [file pone.0102486.s003.tif]
