# Supplementary material for: Oxygen Transport and Stem Cell Aggregation in Stirred-Suspension Bioreactor Cultures
Source: PLoS One. 2014 Jul 17;9(7):e102486. doi: 10.1371/journal.pone.0102486 (PMC4102498; doi:10.1371/journal.pone.0102486)
Supplement: Materials S1 — Supplemental information on cell culture, image analysis, modeling methods and measurements of bioreactor O2 transfer coefficient and HIF1a gene expression levels. (DOCX) [file pone.0102486.s005.docx]

Supplemental Materials

**Oxygen Transport and Stem Cell Aggregation in Stirred-Suspension Bioreactor Cultures**

*J. Wu, M. R. Rostami*, *D.P. Cadavid Olaya, and E. S. Tzanakakis*

**Mouse ESC culture**

Mouse E14Tg2a ESCs (passages 35-50; Mutant Mouse Regional Resource Centers (MMRRC), University of California-Davis, CA) maintained in plates coated with 0.1% gelatin (Sigma-Aldrich, St. Louis, MO) in phosphate buffer saline (PBS) at 5% CO_2_/95% air and 37 °C were adapted to defined serum-free medium (DSFM) as described [[1](#_ENREF_1)]. Dulbecco’s modiﬁed Eagle’s medium (DMEM; Sigma) with 20% KnockOut serum replacer, 0.1 mM nonessential amino acids, 0.055 mM β-mercaptoethanol, penicillin (100 U/mL), streptomycin (100 µg/mL) (all from Invitrogen, Carlsbad, CA), and 1,000 U/mL leukemia inhibitor factor (LIF; EMD Millipore, Billerica, MA). Medium was replaced every day and cells were subcultured every 2-3 days. For passaging, the cells were incubated with TrypLE^TM^ (Invitrogen) in PBS and cell clumps were dissociated into mostly single cells by gentle pipetting. The suspension was spun down and the cell pellet was re-suspended in fresh medium and plated on gelatin-coated dishes. For static aggregate culture, mESCs were plated in Petri dishes and maintained in DSFM.

**Image analysis for calculation of ESC aggregate porosity and tortuosity**

Confocal micrographs of ESC aggregates incubated with FITC-dextran were pre-processed in MATLAB (Mathworks, Natick, MA) with contrast-limited adaptive histogram equalization (CLAHE) to enhance the contrast between cellular matter and pore space. Next, morphological opening was used for background subtraction [[2](#_ENREF_2)] and Gaussian filtering for noise removal. A MATLAB module was written for automatic thresholding of each image. Based on their intensity value after thresholding, pixels were counted either as part of the void area (channels and pores) or cell-occupying space. The total aggregate area was manually segmented in ImageJ and pixels were counted in MATLAB. The porosity was calculated as the ratio of void area to total area for each optical section and averaged over all available sections for each aggregate.

For tortuosity calculations, optical sections after thresholding were skeletonized in ImageJ. Channels in the aggregates were traced with the freehand line tool and the exact path length was determined. The first and last pixels in each path were connected with a straight line (straight line tool) and the corresponding Euclidean distance was computed. Tortuosity was calculated as the ratio of path length to the Euclidean distance [[3](#_ENREF_3)] for at least 20 channels per section and averaged over all available sections for each aggregate (**Fig. 1A**). Tortuosity and porosity values from at least 10 aggregates per culture condition were averaged.

**Transient reaction-diffusion model**

Numerical solutions of Equation 1 in the main text were obtained after discretizing the time and space domains and approximating the temporal derivatives with first-order backward finite differences [[4](#_ENREF_4)]. The integrals were calculated by applying the trapezoidal rule. After subdividing the radius into $n$ equal intervals, Equation 6 yields a set of $n+1$ nonlinear algebraic equations with unknowns the oxygen concentration $C_{i}=C\left(R_{i}\right)$ at each node $R_{i}, i=1\ldots n+1$ along the radius of the aggregate. This system of equations was solved with the Newton-Raphson iterative method. All codes for obtaining numerical solutions were written in FORTRAN.

**Derivation of oxygen transfer coefficient ($\boldsymbol{k}_{\boldsymbol{l}}$) from medium into ESC aggregates**

The boundary condition for the diffusion-reaction equation can be written as:

$D_{t}^{*}\left.\frac{\partial C}{\partial r}\right|_{r=R}=k_{l}\left(C_{M}-C\left(R_{l}\right)\right)$ (Eq. S1)

with aggregates classified according to their size in 7 bins. This number of bins provided proper resolution whereas higher numbers of bins increased the computational load without substantial improvement of the results. The mass transfer coefficient $k_{l}$ was calculated from the Frössling equation [[5](#_ENREF_5)]:

$Sh=2+0.6Re_{p}^{1/2}Sc^{1/3}$ (Eq. S2)

which relates the particle Sherwood number, $Sh=\frac{k_{l}(2\mathrm{R}_{l})}{D}$ with the particle Schmidt $Sc=\frac{v}{D}$, and Reynolds $Re_{p}=\frac{2R_{l}u_{s}}{v}$ numbers. The particle 'slip' velocity $u_{S}$ can be estimated based on the Kolmogorov theory [[6](#_ENREF_6),[7](#_ENREF_7)] and the $Re_{P}$ is expressed in terms of the energy dissipation per unit mass of liquid, $\varepsilon$, as:

$Re_{P}=\left(\frac{16\varepsilon R_{l}^{4}}{v^{3}}\right)^{1/3}$ (Eq. S3)

Calculation of the energy dissipation

$\varepsilon=\frac{P}{V_{M}\rho}$ (Eq. S4)

requires the power input

$P=N_{P}\rho N^{3}d_{imp}^{5}$ (Eq. S5)

and therefore the power number $N_{P}$. General empirical correlations developed by Nagata [[8](#_ENREF_8)] for unbaffled vessels can be used to calculate$N_{P}$.

The power number $N_{P}$ can be calculated [[8](#_ENREF_8)] as

$N_{P}=\frac{\gamma_{1}}{Re_{imp}}+\gamma_{2}\left(\frac{1000+1.2Re_{imp}^{0.66}}{1000+3.2Re_{imp}^{0.66}}\right)^{\gamma_{3}}$ (Eq. S6)

where

$\gamma_{1}=14+\left(\frac{w_{imp}}{d_{V}}\right)\left[670\left(\frac{d_{imp}}{d_{V}}-0.6\right)^{2}+185\right]$ (Eq. S7)

$\gamma_{2}=10^{\left[1.3-4\left(\frac{w_{imp}}{d_{V}}-0.5\right)^{2}-1.14\left(\frac{d_{imp}}{d_{V}}\right)\right]}$ (Eq. S8)

$\gamma_{3}=1.1+4\left(\frac{w_{imp}}{d_{V}}\right)-2.5\left(\frac{d_{imp}}{d_{V}}-0.5\right)^{2}-7\left(\frac{w_{imp}}{d_{V}}\right)^{4}$ (Eq. S9)

Here, $d_{V}$and $w_{imp}$ represent the diameter of the vessel and the width of the impeller, respectively. The Reynolds number for the impeller can be calculated as a function of the agitation rate *N*:$Re_{imp}=\frac{d_{imp}^{2}N}{v}$ (Eq. S10)

**Transformation of the PBE for ESC aggregates**

Although the PBE was expressed in cell/aggregate radius, the density function was transformed to the logarithm of the dimensionless radius ($lnr$). The transformation afforded an extended size range of aggregates to be considered. With the transformation of the density function $n\left(x,t\right)$:

$n\left(x,t\right)=\left[\frac{\partial\left(lnr\right)}{\partial x}\right]n\left(lnr,t\right)=\left(\frac{1}{3x}\right)n\left(lnr,t\right)$ (Eq. S11)

the PBE becomes:

$\frac{\partial n\left(lnr,t\right)}{\partial t}+\frac{\partial\left[n\left(lnr,t\right)\frac{\partial lnr}{\partial t}\right]}{\partial lnr}=r^{3}\int_{0}^{lnr} \frac{n\left(lnr_{c },t\right)}{r_{c}^{3}}n\left(lnr^{'},t\right)K\left(r_{c}\text{|}r'\right)dlnr^{'}-\int_{0}^{\infty} n\left(lnr,t\right)n\left(lnr^{'},t\right)K\left(r\text{|}r'\right)dlnr^{'}$ (Eq. S12)

where the growth term (Gompertz equation) can be written as:

$\frac{\partial\left(lnr\right)}{\partial t}=\frac{a_{G}}{3}ln\left(\frac{M}{r^{3}}\right)$ (Eq. S13)

and the kernel is:

$K\left(r\text{|}r'\right)=k\left(\frac{4}{3}\pi r_{o}^{3}\right)^{7/9}exp\left(-k_{1}\left(\frac{r+r'}{2}\right)^{a}\left(\frac{4}{3}\pi r_{o}^{3}\right)^{\alpha}\right)\left(r+r'\right)^{\frac{7}{3}}$ (Eq. S14)

The term *r_c_* is the dimensionless radius of the aggregate with size *x_c_*. It should be noted that the initial distribution of aggregates was taken as a Gaussian distribution with a mean $lnr=0.5$ and a standard deviation of σ=0.3. Dissociation of ESC colonies before seeding in spinner flasks yielded mostly single cells but also doublets and triplets as starting populations. As such, we assumed the average aggregate size to be that corresponding to nearly 1.6 times of radius of single cells.

The integro-partial differential PBE was solved numerically by finite (backward) differences over equally spaced nodes in the $lnr$ domain. Aggregate size data were smoothened occasionally by the moving average algorithm available in MATLAB without altering the attributes of the measured distributions. The Nelder-Mead method was applied to minimize the difference (Eq. S15) between measured (*n_exp_*) and calculated distributions (*n_model_*) for obtaining values of the kernel parameters. The zeroth and first moments of the cell distribution yield the total number of aggregates and biomass (taking into account the aggregate porosity ε), respectively (Eqs. S16-17).

$\text{Δ}_{n}=\sum^{ } \left(n_{exp}-n_{model}\right)^{2}$ (Eq. S15)

$M_{0}\left(t\right)=n_{0}\int_{x0}^{\infty} n\left(x,t\right)dx$ (Eq. S16)

$M_{1}\left(t\right)=b_{0}\int_{x0}^{\infty} \left(1-\varepsilon\right)xn\left(x,t\right)dx$ (Eq. S17)

where $n_{0}$ and $b_{0}$ are the initial total number of aggregates/cells and biomass, respectively.

**Measurement of oxygen transfer coefficient () from air into bioreactor**

We employed a static degassing method to calculate $\text{k}_{l}^{*}$ values for O_2_ transfer from the air (headspace) to the culture medium in the bioreactor under agitation. Specifically, gas nitrogen was gassed into a 125 ml-spinner flask with 100 ml medium for about 15 min to deplete dissolved O_2_ (DO). Subsequently, the O_2_ recovery profile was measured until the DO exceeded 80% saturation. Linear regression was performed for $\text{ln}\left(C^{*}-C_{M}\right)$ vs. time to acquire the slope term $k_{l}^{*}\cdot a/V_{M}$ (/hr). The value of $k_{l}^{*}$ (cm/hr) can be calculated based on the surface area of the gas-liquid phase and working volume of the bioreactor. The agitation rate was set at 60 rpm. The medium composition was the same as mESC medium (see Materials and Methods) with the addition of 0.1% anti-foaming agent (Sigma-Aldrich, St. Louis, MO) to avoid excessive foaming during the degassing phase.

$V_{M}\frac{dC_{M}\left(t\right)}{dt}=k_{l}^{*}a\cdot\left(C^{*}-C_{M}\left(t\right)\right)$ (Eq. S18)

The measured slope ($k_{l}^{*}\cdot a/V_{M}$) of was 4.87±0.13 /hr yielding a value for $k_{l}^{*}$ of 16.57±0.43 cm/hr (**Fig. S3**).

**Measurement of HIF1α gene expression in ESC aggregates**

*RT-PCR and quantitative PCR*

Total RNA was isolated using Trizol (Invitrogen) according to the manufacturer’s instructions, and reverse transcription (RT) was performed using the ImPromII reverse transcriptase (Promega, Madison, WI).

Quantitative PCR (qPCR) was performed on Bio-Rad CFX96 (Bio-Rad, Hercules, CA) using the Dynamo qPCR Mix (Thermo Scientific) for 40 cycles using the primers in Table S1. All reactions were run in triplicates. Amplification specificity was verified by the melting curve method and gel electrophoresis. Relative gene expression was calculated by normalizing to the expression of endogenous β-actin, using the ∆∆C_t_ method [[9](#_ENREF_9)]. The C_t_ for the housekeeping gene did not vary under different experimental conditions when equal amounts of RNA were used.

**Table S1.** Primers used in qPCR analysis. All primers are shown in 5’-3’ orientation.

| Gene name | Forward primer sequence | Reverse primer sequence |
| --- | --- | --- |
| *ACTB* (human  β-actin) | CTTCCTGGGCATGGAGTCCT | AGGAGCAATGATCTTGATCTTC |
| *HIF1A*  (human HIF1a) | AGGATCAGACACCTAGTCCTTCC | ATCCATTGGGATATAGGGAGCTA |
| *Actb*  (mouse  β-actin) | GCTCTTTTCCAGCCTTCC | GCTCAGGAGGAGCAATGA |
| *Hif1a* (mouse HIF1α) | TGGAACGGAGCAAAAGACTATTA | ACGCTCAGTTAACTTGATCCAAA |

**Supplemental References:**

1. Kehoe DE, Lock LT, Parikh A, Tzanakakis ES (2008) Propagation of Embryonic Stem Cells in Stirred Suspension without Serum. Biotechnol Prog 24: 1342-1352.

2. Wei SF, Yang WH, Hu LL (2008) A background removing method of NM images and its application in the intensity nonuniformity correction methods. 2008 International Special Topic Conference on Information Technology and Applications in Biomedicine, Vols 1 and 2: 326-329.

3. Bullitt E, Gerig G, Pizer SM, Lin WL, Aylward SR (2003) Measuring tortuosity of the intracerebral vasculature from MRA images. IEEE Transactions on Medical Imaging 22: 1163-1171.

4. Tosaka N, Miyake S (1982) Analysis of a nonlinear diffusion problem with Michaelis-Menten kinetics by an integral equation method. Bulletin of mathematical biology 44: 841-849.

5. Frössling N (1938) Uber die Verdunstung fallender Tropfen. Gerlands Gerlands Beitrage zur Geophysik 52: 170-175.

6. Hinze JO (1959) Turbulence; an introduction to its mechanism and theory. Turbulence; an introduction to its mechanism and theory. New York: McGraw-Hill. pp. 183-190.

7. Kolmogorov AN (1941) The local structure of turbulence in incomressible viscous fluids for very large Reynolds numbers. Compt Rend Acad Sci URSS 30: 301.

8. Nagata S (1975) Mixing: Principles and Applications. New York: Wiley.

9. Yuan JS, Reed A, Chen F, Stewart CN (2006) Statistical analysis of real-time PCR data. Bmc Bioinformatics 7.
